# RNA‐Binding Protein IGF2BP2/IMP2 is a Critical Maternal Activator in Early Zygotic Genome Activation

**DOI:** 10.1002/advs.201900295

**Published:** 2019-05-24

**Authors:** Hong‐Bin Liu, Tahir Muhammad, Yueshuai Guo, Meng‐Jing Li, Qian‐Qian Sha, Chuan‐Xin Zhang, Hui Liu, Shi‐Gang Zhao, Han Zhao, Hao Zhang, Yan‐Zhi Du, Kang Sun, Kui Liu, Gang Lu, Xue‐Jiang Guo, Jiahao Sha, Heng‐Yu Fan, Fei Gao, Zi‐Jiang Chen

**Affiliations:** ^1^ Center for Reproductive Medicine Shandong University Jinan 250001 China; ^2^ National Research Center for Assisted Reproductive Technology and Reproductive Genetics Shandong University Jinan 250001 China; ^3^ The Key Laboratory of Reproductive Endocrinology (Shandong University) Ministry of Education Jinan 250001 China; ^4^ CUHK‐SDU Joint Laboratory on Reproductive Genetics School of Biomedical Sciences The Chinese University of Hong Kong Hong Kong China; ^5^ State Key Laboratory of Reproductive Medicine Nanjing Medical University Nanjing 210029 China; ^6^ Life Sciences Institute and Innovation Center for Cell Signaling Network Zhejiang University Hangzhou 310058 China; ^7^ Center for Reproductive Medicine Ren Ji Hospital School of Medicine Shanghai Jiao Tong University Shanghai 200240 China; ^8^ Shanghai Key Laboratory for Assisted Reproduction and Reproductive Genetics Shanghai 200127 China; ^9^ State Key Laboratory of Stem Cell and Reproductive Biology Institute of Zoology Chinese Academy of Sciences Beijing 100864 China

**Keywords:** IGF2, IMP2, infertility, RNA‐binding protein, zygotic genome activation

## Abstract

A number of genes involved in zygotic genome activation (ZGA) have been identified, but the RNA‐binding maternal factors that are directly related to ZGA in mice remain unclear. The present study shows that maternal deletion of *Igf  2bp2* (also commonly known as *Imp2*) in mouse embryos causes early embryonic developmental arrest in vitro at the 2‐cell‐stage. Transcriptomics and proteomics analyses of 2‐cell‐stage embryos in mice reveal that deletion of IMP2 downregulates the expression of *Ccar1* and *Rps14*, both of which are required for early embryonic developmental competence. IGF2, a target of IMP2, when added in culture media, increases the proportion of wild‐type embryos that develop successfully to the blastocyst stage: from 29% in untreated controls to 65% (50 × 10^−9^ m IGF2). Furthermore, in an experiment related to embryo transfer, foster mothers receiving IGF2‐treated embryos deliver more pups per female than females who receive untreated control embryos. In clinically derived human oocytes, the addition of IGF2 to the culture media significantly enhances the proportion of embryos that develop successfully. Collectively, the findings demonstrate that IMP2 is essential for the regulation and activation of genes known to be involved in ZGA and reveal the potential embryonic development‐related utility of IGF2 for animal biotechnology and for assisted reproduction in humans.

## 1 Introduction

Mammalian eggs remain in a quiescent state, but upon fertilization they are reprogrammed into highly specialized totipotent zygotes. This reprogramming sets the embryo onto a developmental course through a series of highly specialized proliferative states and increasingly differentiated stages from 2‐cell‐stage to blastocyst‐stage, ultimately resulting in the development of a new individual.[qv: 1a,b] During early embryonic development, the essential step, which is driven by maternal deposition of RNAs and proteins, is known as zygotic genome activation (ZGA). ZGA is a nuclear reprogramming event that transforms the genome from transcriptional quiescence at fertilization to robust transcriptional activity shortly thereafter. The onset of ZGA varies among species, mainly occurring at the 2‐cell‐stage in mice^2^, ^3^ (9)and at the 4–8‐cell‐stage in humans.^4^ Failure or inappropriate initiation of ZGA in mice leads to developmental arrest, usually at the 2‐cell‐stage and in human, it has been suggested that dysregulated ZGA may contribute to preimplantation pregnancy failure of abnormal embryos.[qv: 5a,b,c]

Many of the proteins known to drive ZGA are maternally encoded, and these are mainly associated with early embryonic development.^6^ Clinically, many in vitro‐matured human oocytes undergo developmental arrest at different stages after fertilization due to genetic variation, inadequate culturing conditions, and/or ZGA defects.[qv: 7a,b,8a,b] Thus, it is important to identify the maternal regulatory factors that are involved in ZGA and to develop strategies for increasing the likelihood of competent embryonic development, which would very likely have a significant impact on improving the success rates of assisted reproductive technologies.

ZGA is notable because of its massive extent of transcription, taking an embryo from a state of little transcription to a state where thousands of genes are being actively transcribed. In contrast, for other transitions, a given cell's global transcription profile remains largely unperturbed, with changes in a few transcription factors often being sufficient to instruct cell fate along specific lineages by activating various subsets of genes. During embryogenesis, the transition from oocyte to embryo is controlled via both transcriptional and epigenetic regulation programs that are known to rely on maternal proteins.[qv: 3,9a,b] Recent studies have documented many maternal proteins in mice, including YAP1, MED13, and TLE6, as having essential functions during ZGA.[qv: 10a,b,11]

The RNA‐binding maternal protein IGF2 mRNA‐binding protein 2 (IGF2BP2), also commonly known as IMP2, belongs to a conserved family of RNA‐binding proteins and is highly expressed in oocytes, embryos, and gonads in mice and in humans.^12^, ^13^ IMP2 has two RNA‐recognizing motifs and four KH domains that control its binding specificity for RNA.[qv: 14a,b,c,d] IMP2 is also known to be essential for RNA processing, and its mRNA transcripts were detected at the 2‐cell‐stage during mice embryogenesis; its expression is widely distributed in developing mouse embryos at E12.5.^15^, ^16^ IMP2 regulates RNA trafficking, the processing of transcripts that encode key subunits in mitochondrial respiratory chain complexes, and IGF2 mRNA translation.[qv: 17a,b,18] IMP2 is directly involved in cancer cell proliferation and laminin β‐2 mRNA translation, and is associated with energy metabolism, the movement of smooth muscles, and the development of type 2 diabetes.[qv: 16,19,20a,b,21] Moreover, recent studies have demonstrated a functional impact of IMP2 in enhancing mRNA stability and translation.^22^ However, any role of the RNA‐binding maternal factor IMP2 in early embryonic development in mice remains unclear.

In this study, we used a maternal *Imp2‐*deletion mouse model to examine the role of maternal IMP2 during ZGA and preimplantation embryonic development. We show that IMP2 is essential for the regulation and activation of genes that are involved in ZGA. Zygotes‐derived from IMP2‐deleted females exhibited embryonic developmental arrest at the 2‐cell stage in vitro, and caused reduced fertility after mating with wild‐type males. Notably, treatment with insulin‐like growth factor 2 (IGF2) (supplementation in the culture medium with IGF2 protein) significantly increased the proportion of embryos that developed into blastocysts. Thus, beyond revealing basic insights about embryonic development and the regulation of ZGA in mammals, our study has implications for developing new technologies for reproductive medicine in humans.

## 2 Results

### 2.1 *Imp2* is Expressed in Mouse Oocytes and Early Embryos

**Figure 1:**
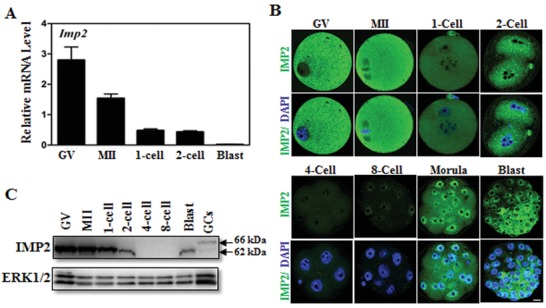
Expression of IMP2 in mouse oocytes and early embryos. A) qRT‐PCR results showing mRNA levels of *Imp2* in mouse oocytes and early embryos. Error bars indicate the SEM. B) Immunofluorescent staining of IMP2 in mouse oocytes and preimplantation embryos. Scale bar, 10 µm. C) Western blot showing IMP2 expression in oocytes and early embryos. GCs, granulosa cells. ERK1/2 is used as the protein loading control.

Protein and mRNA profiles of mouse IMP2 in oocytes and early embryos were determined by Western blotting and qPCR, respectively. We found that transcripts for the mRNA‐binding protein IMP2 were highly expressed in mouse oocytes and early‐stage embryos, with the strongest expression at the germinal vesicle (GV) stage. Its expression was significantly decreased in the subsequent MII oocyte‐stage. Expression was further reduced after fertilization, and was very low by the blastocyst‐stage (**Figure** **1**A).

Immunofluorescence staining with a fluorescence‐conjugated antibody against IMP2 showed that IMP2 is localized in the cytoplasm of oocytes and of preimplantation embryos (Figure 1B). IMP2 expression was evenly distributed in the oocyte stages, but underwent dynamic changes during zygote development: in the morula and blastocyst‐stages, IMP2 had decreased accumulation at the outer edges of blastomeres (Figure 1B). Western blotting further confirmed the expression pattern of the IMP2 protein in oocytes and early embryos (Figure 1C). Collectively, these findings indicate that IMP2 is highly expressed in oocytes and in early‐stages embryos.

**Figure 2:**
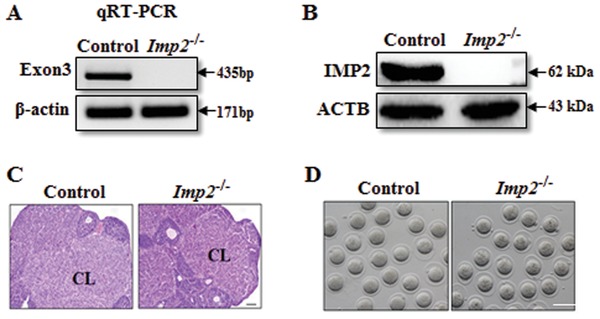
Characterization of *Imp2* mutant mice. A) *Imp2* transcripts were detected in control but not *Imp2^−/−^* ovaries by semiquantitative RT–PCR using β‐actin as the control for the integrity of the RNA samples. Exon 3 was deleted in the *Imp2* knockout strategy. B) IMP2 protein was detected in control but not *Imp2^−/−^* MII lysates by immunoblot using antibodies against IMP2 and ACTB (loading control). Lysate of 100 oocytes in each lane. C) Ovarian histology of control and *Imp2^−/−^* females with hematoxylin and eosin stain. CL, corpus luteum. Scale bar, 100 µm. D) Morphology of MII oocytes from control and *Imp2^−/−^* females after superovulation at postnatal day 23. Females (*n* = 10) were used for each genotype. Scale bar, 100 µm.

To investigate the physiological function of IMP2, a conventional *Imp2*‐knockout mouse was generated by flanking exons 3 and 4 of the *Imp2* locus (Figure S1A, Supporting Information). No *Imp2* transcript expression was detected from *Imp2^−/−^*‐derived ovaries and from MII‐stage egg lysates (**Figure** **2**A,B). Having generated *Imp2*‐knockout mice and subsequently confirmed that no *Imp2* mRNA transcripts or IMP2 protein could be detected in knockout mice, we next examined the role of IMP2 in folliculogensis and in oocyte maturation. *Imp2^−/−^* females had normal folliculogenesis and corpora lutea, and were indistinguishable from control mice (Figure 2C). The numbers and morphologies of MII‐stage oocytes derived from *Imp2^−/−^* female mice showed no significant differences compared with control mice (Figure 2D and Figure S1B, Supporting Information). These findings suggest that *Imp2* is not required for oocyte maturation.

### 2.2 Deletion of Maternal *Imp2* Results in Early Embryonic Developmental Arrest

**Figure 3:**
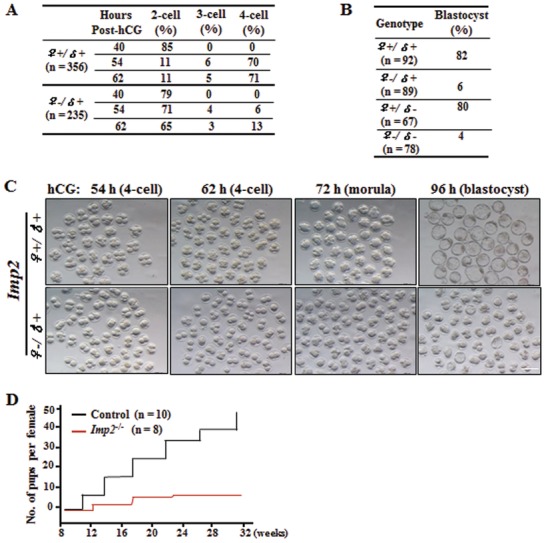
Maternal deletion of IMP2 causes impaired early embryogenesis. A) Maternal IMP2 deletion inhibits early embryonic development. *n* > 10 mice for each genotype. Data are presented from three independent experiments, and total numbers of zygotes (*n*) of the two genotypes in the analysis are indicated. B) Maternal IMP2 deletion causes impaired blastocyst formation. Numbers of embryos (*n*) flushed in vivo are indicated. *n* > 5 mice for each genotype. Data are presented from three independent experiments, and total numbers of embryos (*n*) of the two genotypes in the analysis are indicated. C) Morphology of *Imp2* female embryos cultured in vitro after mating with WT males. Embryonic development was monitored over the indicated time frame after hCG administration. Scale bar, 100 µm. D) Cumulative numbers of pups per female during the defined time period. *n* > 7 mice for each genotype.

To investigate the role of IMP2 in early embryonic development, we monitored female germline cells at different stages of oocyte development in control and *Imp2^−/−^* female mice. No IMP2 expression was detected at the oocyte‐stage in *Imp2^−/−^* female mice (Figure S2A, Supporting Information). To investigate the contribution of *Imp2* to embryonic development, control and *Imp2^−/−^* females were mated with WT males. After successful mating, control female‐derived zygotes (*Imp2* ^♀+/♂+^) and *Imp2^−/−^* female‐derived zygotes (*Imp2* ^♀−/♂+^) were collected, and these were cultured in KSOM medium. No significant differences were observed in the development or morphology of zygotes or 2‐cell‐stage embryos (**Figure** **3**A and Figure S2B, Supporting Information). However, *Imp2^−/−^* female‐derived embryos (*Imp2* ^♀−/♂+^) showed delayed progression through cell division at 54 hours post‐hCG treatment, and >90% of them eventually arrested at the 2‐cell‐stage, and exhibited impaired embryonic developmental configurations (Figure 3A,C). In vivo blastocyst flushing results indicated that only 6% of the *Imp2* ^♀−/♂^ **^+^** embryos developed into the blastocyst‐stage, as compared to 82% for the control embryos (Figure 3B). Most *Imp2* ^−/−^ embryos died prior to compaction (>80%) or fragmented into cytoplasmic blebs (Figure S2D, Supporting Information). Note that we observed consistent results for embryonic development when in vivo flushing of blastocyst at E3.5 was used after successful mating with males compared with obtained in vitro culture blastocyst proportion in *Imp2^−/−^* females (Figure 3B and Figure S2C, Supporting Information). To determine the role of an intact paternal *Imp2* allele, an *Imp2*‐knockout male germline was developed. *Imp2^−/−^* males with normal fertility and spermatogenesis were used for breeding with control and *Imp2^−/−^* females. Pregnant females were sacrificed at 3.5 days post coitus, and blastocysts were flushed. No significant effects were observed in blastocyst formation percentage after deletion of paternal *Imp2* (Figure 3B). These findings suggest that *Imp2* may be required for preimplantation embryonic development.

We further investigated the fertility of control and *Imp2^−/−^* females older than 5 weeks when mated with WT adult males over a period of 6 months. *Imp2^−/−^* females were subfertile, producing significantly fewer pups than control females (Figure 3D). In the first one or two litters, *Imp2^−/−^* females produced three or four pups, but this number gradually decreased until the mice became infertile. *Imp2^−/−^* females produced accumulated 8.57 ± 1.3 pups per female, whereas control mice produced 49 ± 2.8 pups per female (Figure 3D). Thus, *Imp2* functionally contributes to female fertility in mice.

### 2.3 Deletion of *Imp2* Results in Extensive Downregulation of Transcription during Zygotic Genome Activation

**Figure 4:**
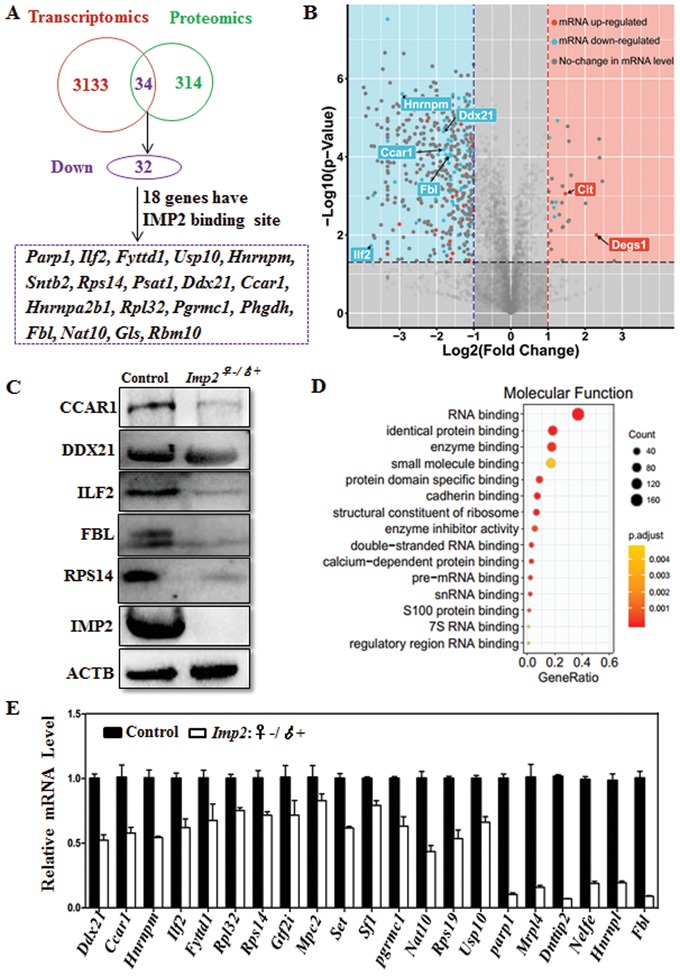
Deletion of *Imp2* results in extensive downregulation of transcription during zygotic genome activation. A) Schematic diagram showing the late 2‐cell‐stage, control embryos and *Imp2‐*knockout embryos (*Imp2^♀−/♂+^*) for RNA sequencing (20 embryos per group, 3 replicates) and proteomic analyses (330 embryos per group, 3 replicates). B) Volcano plot showing the downregulated and upregulated genes in 2‐cell‐stage *Imp2‐*knockout embryos in fold change (*x*‐axis) and statistical significance (−log10 of the *p* value, *y*‐axis). Different dots indicate the transcription change, while the background color represents the protein change. The red dots indicate upregulated genes, the blue dots indicate downregulated genes, and the gray dots indicate no change in transcription. *Cit* and *Degs1* are upstream genes of *Imp2*. C) Western blot of 2‐cell‐stage embryos from control and *Imp2^−/−^* female mice probed with antibodies against CCAR1, DDX21, ILF2, FBL, RPS14, IMP2, and ACTB. D) Gene ontology analysis of the downregulated genes in *Imp2* ^♀^ *^−^* ^/♂+^ embryos compared with control embryos at the 2‐cell‐stage. E) Quantitative real‐time PCR (qRT‐PCR) analysis showing the expression of transcripts in control and *Imp2* ^♀^ *^−^* ^/♂+^ embryos at the 2‐cell‐stage. Error bars indicate the SEM.

During the growth of oocytes, meiotic progression in transcriptionally silent oocytes is coordinated with translation of some maternal transcripts,^23^ and this synchronization is essential for the maturation of oocytes and for supporting early embryonic preimplantation development.^23^, ^24^ Therefore, seeking to identify genes potentially regulated by *Imp2*, we conducted an RNA‐seq analysis of GV‐stage and MII‐stage oocytes from control and *Imp2^−/−^* mice. The results indicated that control and *Imp2^−/−^* oocytes exhibited similar transcriptomes at GV‐stage and MII‐stage (File S1, Supporting Information). Phenotypic analysis of in vitro cultures showed that *Imp2‐*deleted female oocytes arrested at the 2‐cell‐stage; therefore, seeking to identify genes regulated by *Imp2* in embryos, we performed RNA‐seq and mass spectrometry‐based proteomic analysis of 2‐cell‐stage embryos‐derived from control and *Imp2^−/−^* females (**Figure** **4**A). Following analysis using the Cufflinks data analysis pipeline, we found 1526 upregulated and 1607 downregulated genes out of a total of 3133 transcripts that were differentially expressed in *Imp2^−/−^* embryos as compared to those from control females (Figure 4A). However, proteomics analysis identified 32 upregulated proteins and 282 downregulated proteins out of a total of 314 proteins (Figure 4A).

2‐cell‐stage data from the transcriptome and proteome analyses were merged to further identify the downregulated genes (Figure 4A). A total of 34 genes were identified after merging the RNA‐seq and proteomic data of 2‐cell‐stage embryos, however; 32 were downregulated out of a total of 34 genes common to both datasets (Figure 4A and Table S4, Supporting Information). A total of 18 downregulated genes were found to have *Imp2* binding sites after comparing RNA‐seq and proteomic merged genes analyses results with data from photoactivatable ribonucleoside‐enhanced crosslinking and immunoprecipitation^25^ (Figure 4A, File S2, Supporting Information). We found the genes obtained after RNA‐seq and proteomic merged data indicated reduced transcriptional and translational level after *Imp2* knockout (Figure 4B). Western blotting of 2‐cell‐stage embryos with antibodies against CCAR1, ILF2, FBL, RPS14, and IMP2 proteins revealed consistent trends in downregulation as those seen in the merged RNA‐sequencing and proteomics datasets (Figure 4C). The only detected exception was the DDX21 protein expression in *Imp2^−/−^* female‐derived 2‐cell‐stage embryos (Figure 4C). Gene ontology (GO) analysis revealed that many of these downregulated genes had functional annotations related to RNA‐binding and protein‐binding activities (Figure 4D). The mRNA level of these downregulated genes in control and *Imp2^−/−^* 2‐cell‐stage embryos were evaluated using qPCR, and these were consistent with the RNA‐seq and proteomics merged datasets (Figure 4E).

**Figure 5:**
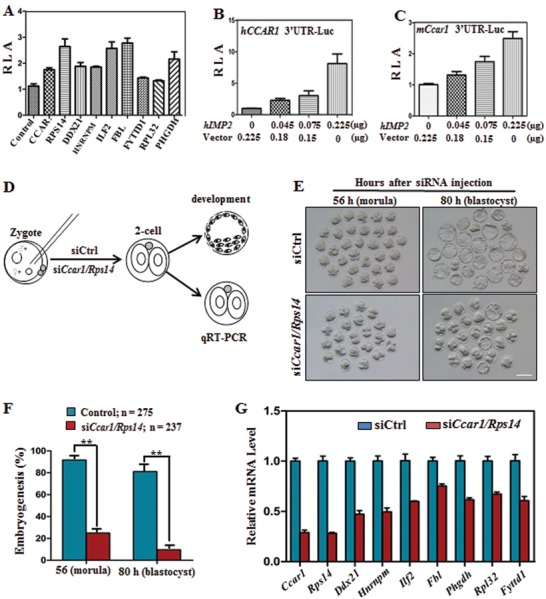
*Ccar1* and *Rps14* are key target genes of IMP2 that mediate early embryonic developmental potential. A) Luciferase reporter activity of the indicated downregulated genes containing IMP2 binding sites. RLA, relative luciferase activity. Error bars indicate the SEM. B,C) Luciferase reporter activity of *hCCAR1* in response to IMP2 in a dose‐dependent manner (B), and luciferase reporter activity of downstream *mCcar1* in response to IMP2 (C). Error bars indicate the SEM. At least three independent experiments were performed for mean of each value. D) Schematic diagram showing the microinjection of early mouse zygotes and subsequent embryo analysis at the molecular and developmental level. E) Blastocyst development is defective after injecting siRNAs targeting the *Ccar1* and *Rps14* genes at the indicated times compared with control siRNA. Scale bar, 100 µm. F) Quantification of morula (56 h) and blastocyst (80 h) formation after injecting control siRNA or siRNAs targeting *Ccar1* and *Rps14*. The numbers of embryos (*n*) analyzed are indicated. Error bars indicate the SEM. **p < 0.01, Student's *t*‐test. G) qRT‐PCR results showing the expression of IMP2 target genes in 2‐cell‐stage embryos after *Ccar1/Rps14* deletion in zygotes. Error bars indicate the SEM. *p < 0.05, Student's *t*‐test.

Seeking to confirm direct interactions between IMP2 and these putative target genes, we compared our 2‐cell‐stage embryos proteomics data with IMP2 photoactivateable ribonucleoside‐enhanced cross‐linking and immunoprecipitation, and selected 9 genes out of total 18 genes having IMP2 binding sites in order to monitor the luciferase reporter activity in response to IMP2 (**Figure** **5**A). To determine whether IMP2 alters transcriptional activity through binding to the 3'‐UTR of target genes, a luciferase reporter assay was performed in a dose‐dependent manner on these selected genes in human and mice. The transcriptional profile was monitored with dual luminescence assays based on luciferase activity in relation to increasing amounts of IMP2. In luciferase reporter assay, promotors of target genes in human and mice elicited strong responses to IMP2 in dose‐depended stimulation (Figure 5B,C and Figure S3A–G, Supporting Information).

Among them, *Ccar1* and *Rps14* were found to be the target genes for IMP2. The induction of these two genes might be required for early embryonic development. To verify the role of these two genes as target genes for IMP2, combined knockdown of *Ccar1* and *Rps14* experiment was performed in WT zygotes (Figure 5D). Knockdown of *Ccar1* and *Rps14* decreased embryonic development compared to WT embryos, which were here used as controls (Figure 5E,F). Reduced mRNA expression was observed in *Ccar1* and *Rps14*‐depleted embryos as indicated by qRT‐PCR analysis (Figure 5G). Collectively, these results suggest that deletion of *Imp2* causes downregulation of genes transcription during ZGA and indicates that *Ccar1* and *Rps14*, target genes of *Imp2*, might be essential for the regulation of embryonic development.

*Imp2* mRNA was injected into *Imp2^−/−^* female‐derived zygotes that were cultured in vitro in G‐1 and G‐2 medium. The *Imp2^−/−^* female‐derived zygotes were partially rescued from the 2‐cell‐stage arrest, and 33% *Imp2^−/−^* embryos progressed to the blastocyst stage, as opposed to 11% percent of the untreated *Imp2^−/−^* embryos (Figure S2G, Supporting Information). These results imply that IMP2 functions specifically during the oocyte‐to‐embryo transition.

### 2.4 *Imp2* Deletion Disrupts Both Transcriptional and Translational Machinery in 2‐Cell‐Stage Embryos

**Figure 6:**
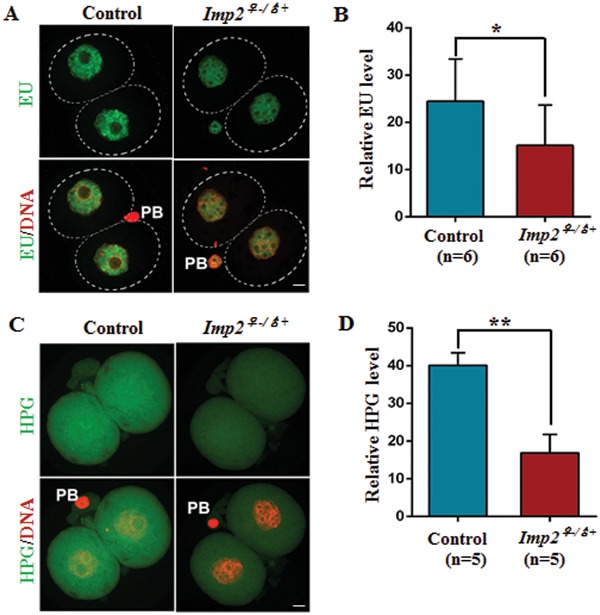
Deletion of IMP2 disrupts the transcriptional and translational activity in 2‐cell‐stage embryos. A) Confocal image showing newly synthesized RNA by EU staining in control and 2‐cell‐stage *Imp2^−/−^* female embryos. Scale bar, 20 µm. B) Quantification of the fluorescence in newly synthesized RNA in control and *Imp2^−/−^* female 2‐cell‐stage embryos by EU incorporation. More than 10 embryos were observed for each genotype with six replicates. *n* = 6 mice for each genotype. Error bars indicate the SEM. *p < 0.05, Student's *t*‐test. C) Confocal image indicating the protein synthesis in control and *Imp2^−/−^* female 2‐cell‐stage embryos incorporating HPG. Scale bar, 20 µm. D) Quantification of the fluorescence in nascent protein synthesis by HPG incorporation in control and *Imp2^−/−^* female 2‐cell‐stage embryos. More than 10 embryos were observed for each genotype with six replicates. *n* = 5 mice for each genotype. Error bars indicate the SEM. **p < 0.01, Student's *t*‐test.

The gene expression reprogramming that is required during early embryonic preimplantation development coincides with changes in chromatin structure that are associated with RNA synthesis.[qv: 26a,b,27] To determine the role of IMP2 in transcriptional activity, we used 2‐cell‐stage embryos samples of both genotypes (control and *Imp2^−/−^*) for 5‐ethynyl uridine (EU) incorporation assay. EU, which is an alkyne‐modified nucleotide, can be actively incorporated into nascent RNA when incubated with the oocytes and embryos, and the EU signal is indicative of total RNA synthesis in 2‐cell‐stage embryos. The intensity of the EU signals was greatly decreased in *Imp2^−/−^* female‐derived 2‐cell‐stage embryos compared with control embryos (**Figure** **6**A,B), thus revealing defects in transcriptional activity.

To test whether *Imp2* deletion affects total protein synthesis during ZGA, 2‐cell‐stage embryos were incubated in culture medium supplemented with 50 × 10^−6^ m HPG (L‐homopropargylglycine) for 2 h. HPG signal intensity, which is indicative of translational activity, was two times lower in IMP2‐depleted, 2‐cell‐stage embryos compared to control embryos (Figure 6C,D). Taken together, our results indicate that the transcriptional and translational activity that is essential for gene expression during embryonic growth is IMP2 dependent.

### 2.5 IGF2 Supplementation Increases the Proportion of Zygotes that Eventually Develop into Blastocysts

**Figure 7:**
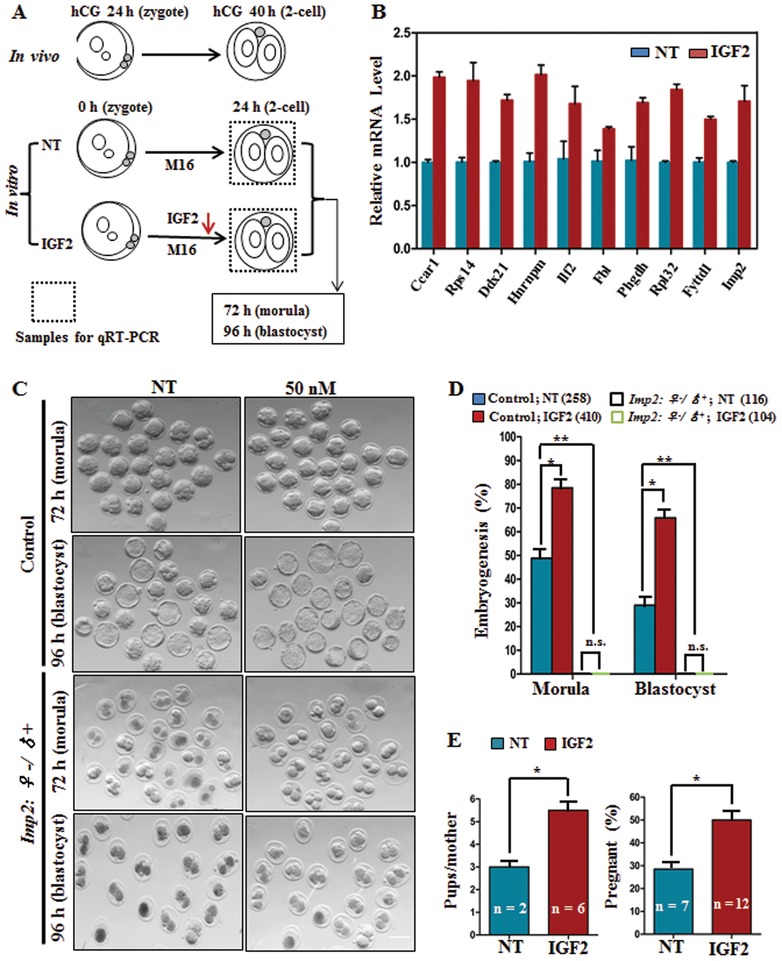
IGF2 supplementation increases the proportion of zygotes which eventually develop into blastocysts. A) Schematic diagram showing IGF2 treatment of early embryos in M16 medium in vitro. NT: no treatment. B) IGF2 treatment triggers the expression of IMP2 target genes in 2‐cell‐stage embryos. Error bars indicate the SEM. *p < 0.05, Student's *t*‐test. C,D) Morphology (C) and quantification (D) showing that IGF2 treatment increases the early embryonic developmental efficiency of control embryos but has no effect on *Imp2* ^♀−/♂^ **^+^** embryos. The numbers of embryos (*n*) analyzed are indicated. *n* > 15 mice for both genotypes. Error bars indicate the SEM. *p < 0.05 and **p < 0.01, Student's *t*‐test. NS: not significant; NT: no treatment. Scale bar, 100 µm. E) Embryo transfer experiments showing greater rates of embryo development to term after IGF2 treatment. The number of pups per mother is on the left, and the percentage of pregnant mice is on the right. *n* represents the number of pregnant females on the left and the total number of foster mothers used on the right. Error bars indicate the SEM. *p < 0.05, Student's *t*‐test. NT: no treatment.

M16 is a widely used culture medium, but it reduces the rate of embryonic development into the morula and blastocyst‐stages.^28^ And thus different growth factors have been added to the culture medium to improve early embryonic growth.^11^, ^29^ Previously, IGF2 has been used for the maturation of porcine oocytes.^30^ To determine the functional role of IGF2 in embryonic development, control and *Imp2^−/−^* female‐derived zygotes were cultured in M16 medium with or without IGF2 (**Figure** **7**A). IGF2 treatment promoted the expression of *Imp2* downstream genes in cultured embryos (Figure 7B), and adding IGF2 to the culture medium improved the development rate of control embryos (*Imp2* ^♀+/♂+^), but no effect was observed in *Imp2^−/−^* female‐derived embryos (*Imp2* ^♀−/♂+^) (Figure 7 C,D).

To further investigate the developmental fate of IGF2‐treated embryos in vivo, embryo transfer experiment was performed. In an experiment related to embryo transfer, 12 foster mothers for IGF2‐treated embryos and 7 for nontreated control embryos were used as the recipients. Foster mothers receiving IGF2‐treated embryos delivered more pups per female, and their pregnancy rate was also significantly greater than females who received nontreated control embryos (Figure 7E and Figure S2F, Supporting Information). These results suggest the potential of IGF2 to improve the embryonic developmental competency in control embryos, but have no effect on *Imp2^−/−^‐*derived embryos.

### 2.6 IMP2 Positively Regulates the Expression of IGF2

Next, the mRNA expression level in control and *Imp2^−/−^* female‐derived oocytes and embryos were determined. qPCR was performed to observe IGF2 mRNA expression levels in control and *Imp2^−/−^* female‐derived GV‐stage, MII‐stage, and 2‐cell‐stage embryos. Compared with control female‐derived oocytes and embryos, a significantly reduced IGF2 mRNA expression level was observed in *Imp2^−/−^*‐derived GV‐stage, MII‐stage oocytes, and 2‐cell‐stage embryos (Figure S4A, Supporting Information). To determine the IGF2 protein level in control and *Imp2^−/−^* female, the GV‐stage oocytes from both genotypes were collected and cultured in M16 medium supplemented with 2 × 10^−6^ m milrinone to inhabit the maturation for 12 h. The IGF2 protein level in culture medium containing control and *Imp2^−/−^* female‐derived GV‐stage oocytes was measured using an ELISA kit (R & D system, MG200). The results showed twofold lower IGF2 level in medium containing *Imp2^−/−^* female‐derived GV‐stage oocytes compared with control, GV‐stage oocytes culture medium (Figure S4B, Supporting Information). These results indicated the reduced IGF2 protein level in *Imp2^−/−^* female‐derived oocytes.

To verify the direct relationship between IMP2 and IGF2, we next identified the 5′‐UTR binding sites of IMP2 in IGF2 in the cross‐linking immunoprecipitation database (http://starbase.sysu.edu.cn/rbpClipRNA.php?source=mRNA). This analysis identified three binding sites at the IGF2 L3 locus on chr11 (Table S5, Supporting Information). The IGF2 L3 5′‐UTR was cloned, and a dual luciferase reporter assay (Genecopoeia, #SPGA‐G010) was performed in vitro in HEK293 cells. The results indicate that IMP2 binds to the IGF2 L3 5′‐UTR and enhanced the luciferase activity of IGF2 by twofold compared to a negative control (Figure S4C, Supporting Information). The regulatory function of IMP2 on IGF2 expression might be the promotion of translation initiation. These results strongly suggest that IMP2 binds with IGF2 and regulates its expression.

### 2.7 IGF2 Supplementation Improves Human Early Embryos Development In Vitro

**Figure 8:**
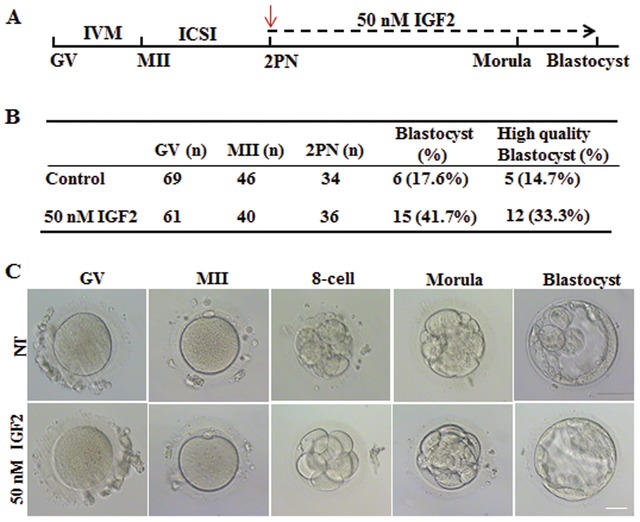
IGF2 supplementation improves human early embryos development in vitro. A) Time line of human oocyte maturation to early embryo growth up to the blastocyst stage, highlighting critical times between the stages and the predicted in vitro culture development in medium with and without IGF2 after intracytoplasmic sperm injection. Red arrows indicate the time duration of IGF2 treatment from zygote to blastocyst formation. B) Improved blastocyst formation of human embryos after IGF2 treatment. Total numbers of zygotes (n) used are indicated. C) Morphology of embryos after in vitro culture with or without IGF2 in the culture medium. Scale bar, 100 µm.

In mice, after verifying the increased proportion of morula and blastocyst with IGF2 supplementation in culture medium, we examined the effect of IGF2 supplementation in culture medium on human embryos in vitro development. Zygotes were cultured in medium with or without 50 × 10^−9^ m IGF2 after in vitro maturation and intracytoplasmic sperm injection of oocytes (**Figure** **8**A). No significant difference was observed in the percentage of 8‐cell‐stage embryos between the two groups, but increased blastocyst formation (41.7%) was observed in the IGF2‐treated embryos compared to the control embryos (17.6%) (Figure 8B). Moreover, the blastocyst quality was assessed by a clinician with extensive experience and a larger proportion of high‐quality blastocysts was observed from cultured embryos treated with IGF2 compared with controls (Figure 8B,C). Thus, adding IGF2 to the culture medium increased the rate of blastocyst formation along with improved quality, and this suggests the IGF2 has potential to improve the in vitro embryos developmental proportion in human assisted reproduction techniques.

## 3 Discussion

It has been reported that many maternal effect proteins including YAP1, BTG4, RNF114, NLRP2, and OCT4 are involved and activated during ZGA and deletions of these protein cause most embryos to arrest at the 2‐cell‐stage or before compaction.[qv: 11,31a,b,c,d] but only a limited number of RNA‐binding maternal proteins in mice that are involved in the oocyte‐to‐embryo transition have been reported previously.^32^ The functional activity and involvement of IMP2 in type 2 diabetes, energy metabolism, and cancer development have been reported previously.^16^, ^19^, ^21^ However, the contribution of maternal *Imp2* mRNA and protein during mouse embryogenesis has remained to be studied. Here, we generated an *Imp2‐*knockout mouse model and then set out to understand the zygote‐derived role of IMP2 on ZGA and early embryonic development. We first conducted a detailed phenotype assessment of preimplantation embryos development‐derived from *Imp2^−/−^* female in vivo and in vitro and observed 2‐cell‐stage arrest after knockout of *Imp2* in vitro. Furthermore, downregulated genes were identified in 2‐cell‐stage embryos‐derived from control and *Imp2^−/−^* female via transcriptomic and proteomic analyses, and *Ccar1* and *Rps14* were found to be the direct target genes of *Imp2* that are required for early embryonic development. In addition, the role of IMP2 in RNA and protein synthesis during ZGA in 2‐cell‐stage embryos from control and *Imp2^−/−^* female was determined. Finally, the effect of IGF2 supplementation in cultured media for improving the proportion of embryos that developed into blastocysts was observed in mice and human, and the direct relationship between IMP2 and IGF2 was confirmed by a luciferase reporter assay.

IMP2 was originally identified as RNA‐binding protein capable of binding IGF2 mRNA,^12^ but later reports suggested that it also targets number of other mRNA transcripts. The IMP2 indicated multitargeted physiological functions. IMP2 expression pattern peaks at embryonic day 16.5 in mouse gonads.^13^ However, our study showed that it is dispensable for folliculogenesis and oocyte maturation. During embryonic preimplantation development, programmed waves of gene activation might serve to satisfy the embryo's requirement for growth because ZGA transcripts and protein products seem to be required for the progression of embryonic development.[qv: 33a,b] Thus, inhibition of genome transactivation due to ZGA defects might be the cause of embryonic developmental arrest as we observed in our study. It has been reported that embryos associated with impaired transcriptional machinery exhibit severe embryonic developmental defects during ZGA.[qv: 27,34a,b] Previous reports have also shown that gene expression regulation in mammals depends upon the translational machinery, which is directly involved in oocyte and embryo development.[qv: 35a,b,c] Thus, IMP2 deletion causes inhibition of genome activation as well as defects in transcriptional and translational machinery, which might be a reason for the observed impairment of embryonic development.

Growth factors have been added to the culture medium to enhance the developmental competency of oocytes and embryos in different species, including humans and mice.^11^, ^36^ Previously, IGF2 has been shown to improve the maturation rates of porcine oocytes,^30^ but the role of IGF2 in embryonic developmental competency during in vitro culture was demonstrated in our current study. After number of trials with different IGF2 concentrations, we found that adding 50 × 10^−9^ m IGF2 to the MI6 medium was the optimum concentration for improving the proportion of control embryos that developed into blastocysts (Figure S2E, Supporting Information). Cells supplemented with a plasmid expressing IGF2 have been shown to exhibit autocrine effects and to undergo increased cell proliferation without the addition of growth factors to the culture medium.^37^ Moreover, mouse embryonic fibroblast cell proliferation is increased by the addition of IGF2.^38^ A clinical study was reported that the oocytes retrieved from follicular fluid containing greater concentrations of IGF2 exhibit enhanced maturation and improved overall embryonic development; the study also noted that increased IGF2 levels were correlated with embryo scores in humans.^39^ These studies support the notion of a growth‐promoting potential for IGF2 in mice and also in human.

The growth‐promoting activity of IGF2 is mediated by at least three structurally related receptors, IGF1R, IGF2R, and insulin receptor.^40^, ^41^ IMP2 interacts with IGF2, which ultimately upregulates the translation of IGF2 and ensures the availability of IGF2 to bind to IGF1R, which induces phosphorylation and subsequent activation of the PI3K/Akt signaling pathway and results in protein translation, embryo development and cell proliferation.^19^, ^41^ It has been reported that *Imp2* promotes IGF2 mRNA translation.^18^ and the abundance of IGF2 is directly associated with overexpression of *Imp2*, which correlates with cell proliferation.^19^ Overexpression of *Imp2* increases the level of IGF2 protein in cell lines, and silencing of *Imp2* decreases these levels.^19^ Previous studies have demonstrated that IMP2 directly binds to the IGF2L3 5′‐UTR and shows an abundance of luciferase activity in a dose‐dependent manner.^21^ The inhibition of IGF2 delays cell growth and abrogates the proliferation‐promoting effect of *Imp2*.^19^

Clinically, many in vitro matured human oocytes showed developmental arrest at different stages after fertilization, owing to suboptimal culturing conditions.^8^ Addressing the issue of culture conditions, IGF2 would improve both human and mouse embryonic growth in vitro and might prove to be an integral part of assisted reproductive technologies. Furthermore, in clinics the embryonic developmental competence of immature oocytes derived from patients suffering reproductive dysfunction could perhaps be improved with IGF2 supplementation in culture media. Although IGF2 apparently led to improve in vitro embryonic development in humans in our IGF2 supplementation experiment in culture media but its functional roles are known to be very complicated, so further investigation regarding pregnancy outcomes and safety evaluation will be required to assess the feasibility of any IGF2‐based clinical interventions.

## 4 Conclusion

Together, we provide compelling evidence that IMP2 is essential for the regulation and activation of genes that are involved in ZGA and show that supplementing culture media with IGF2 can increase the proportion of embryos that developed into blastocyst from 29% in untreated controls to 65% (50 × 10^−9^ m IGF2) in mice and 17.6% untreated controls to 41.7% (50 × 10^−9^ m IGF2) in human.

## 5 Experimental Section

*Establishment of Knockout Mice*: The *Imp2*‐knockout mouse line was constructed in compliance with the guidelines of the animal care and research committee of Shandong University. This study was approved by the Institutional Review Board (IRB) of Reproductive Medicine of Shandong University (2018, IRB. No. 3). To generate conventional knockout mice, exons 3 and 4 of the *Imp2* were flanked with LoxP sites (Figure S1A, Supporting Information), then *Imp2^flox/flox^* mice were crossed with Vasa‐Cre (also known as Ddx4‐Cre, germ cell specific expression) transgenic mice to obtain *Imp2^flox/+^*;Vasa‐Cre mice that were intercrossed to generate conventional knockout mice. All mutant mouse strains had an ICR and C57BL/6J mixed background. The primers used for genotyping are shown in Table S2 in the Supporting Information.

*Oocyte/Embryo Collection and Microinjection*: Pubertal female mice (24–28 days old) were superstimulated with 5 IU pregnant mare's serum gonadotropin (PMSG) followed by 5 IU human chorionic gonadotrophin (hCG) after 44 h. Oocytes were collected and cultured in small drops of M16 medium (M7292; Sigma‐Aldrich) and were covered with mineral oil and maintained in 5% CO_2_ at 37 °C. For collection of zygotes and embryos, control and *Imp2^−/−^* females were mated with adult WT males post‐hCG injection. Zygotes and embryos were collected from the oviducts and uteri at the indicated time points after hCG administration. For microinjection, mRNAs were transcribed in vitro with the mMESSAGE mMACHINE SP6 transcription kit (Invitrogen, AM1450). siRNA was obtained from RiboBio, and the sequences are shown in Table S2 in the Supporting Information.

*Zygote Culture, Embryo Transfer, and Fertility Assessment Test*: Control and *Imp2^−/−^* females zygotes were cultured in KSOM medium (Sigma‐Aldrich) at 37 °C in 5% CO_2_ for observing their embryonic developmental potential. For the microinjection‐related experiment, the embryos were cultured in G‐1 and G‐2 media (Sigma‐Aldrich).

For the IGF2 protocol, zygotes were cultured in M16 medium with or without 50 × 10^−9^ m IGF2 (CF61, Novoprotein). Embryonic development and morphology were examined with a stereomicroscope (Nikon SMZ1500).

In an experiment related to embryo transfer, blastocysts obtained with and without IGF2 treatment were used. A total of 19 pseudopregnant Kunming female mice were used as the recipients (16 embryos were transferred to the uterus of each mouse). The pregnancy rates to term and the litter sizes were recorded.

To assess the fertility, control and *Imp2^−/−^* females were crossed to adult WT males for a period of 6 months. 3 females of each genotype (older than 5 weeks) were assigned per cage with one adult WT male. Less than 10 females were allocated for each genotype for the experiment. Fertility was assessed as the total number of pups per female during 6‐month test period.

*Culture of Human Zygotes with IGF2 Treatment*: Spare human GV oocytes were collected and matured in vitro in 6% CO_2_, 5% O_2_, and 90% N_2_ at 37 °C. After maturation, MII oocytes were used for the ICSI protocol. Zygotes with intact morphology were allocated to the control and experimental groups. Zygotes were cultured with or without 50 × 10^−9^ m IGF2 (CF61, Novoprotein) and incubated in 6% CO_2_, 5% O_2_, and 90% N_2_ at 37 °C. The assessment of embryonic development and embryo quality was performed by a clinician with extensive experience, and photomicrographs were taken at different stages of development. Written, informed consent was obtained from all the candidates before participation.

*RNA Extraction and qRT‐PCR Validation*: The RNeasy mini kit (Qiagen) was used for the extraction of total RNA following the manufacturer's instructions. Genomic DNA was removed by digesting with RNase‐free genomic DNA eraser buffer (Qiagen), and cDNA was obtained by reverse transcription of RNA using PrimeScript reverse transcriptase (Takara). Power SYBR Green Master Mix (Takara) was used on a Roche 480 PCR system for qRT‐PCR analysis. The mRNA level was calculated by normalizing to the endogenous mRNA level of actin (internal control) using Microsoft Excel. The qRT‐PCR reactions were performed in triplicate for each experiment using gene‐specific primers. Primer sequences are shown in Table S2 in the Supporting Information.

*RNA Sequencing and Quantitative Proteomic Analysis*: For RNA sequencing, GV‐stage, MII‐stage, and late 2‐cell‐stage embryos were collected from control and *Imp2^−/−^* females (20 embryos per group, 3 replicates). The RNA sequencing protocol was carried out as described previously by Fan.^11^ Briefly, total RNA was isolated from oocytes/embryo samples using the RNAeasy mini kit (Qiagen) according to the manufacturer's protocols. mRNA‐RFP was added to calculate the mRNA copy number. The NEB Next Ultra RNA library prep kit for Illumina was used for generating the sequencing library using extracted total RNA. The library was sequenced by Hiseq X Ten, and the RNA‐sequencing reads were aligned to the *Mus musculus* UCSC mm9 reference genome using the Tophat software (http://tophat.cbcb.umd.edu/). Proteomic analysis was carried out using a protocol as described previously by Guo et al.^42^ Briefly, embryos at the late 2‐cell‐stage were collected from control and *Imp2^−/−^* females (330 embryos per group, three replicates). Embryos were lysed in protein extraction buffer consisting of 75 × 10^−3^ m NaCl, 50 × 10^−3^ m Tris (pH 8.2), 8 m urea, 1 × 10^−3^ m NaF, 1% (v/v) EDTA‐free protease inhibitor cocktail, 1 × 10^−3^ m β‐glycerophosphate, 1 × 10^−3^ m sodium orthovanadate, 10 × 10^−3^ m sodium pyrophosphate, and 1 × 10^−3^ m PMSF. Lysates were centrifuged at 40000 × *g* for 1 h, and the Bradford assay was used to measure the protein concentration. Cysteines were reduced with 5 × 10^−3^ m DTT for 25 min at 56 °C followed by alkylation in 14 × 10^−3^ m iodoacetamide for 30 min at room temperature. Samples were digested overnight with trypsin using an enzyme‐to‐substrate ratio of 1:200, and the resulting peptides were divided into aliquots. The samples were then subjected to tandem mass tags labeling. Aliquots of the same samples were combined, lyophilized, and resuspended in buffer A (10 × 10^−3^ m ammonium acetate, pH 10) to a final volume of 110 µL and then loaded onto an XBridgeTM BEH130 C18 column (2.1 mm × 150 mm, 3.5 µm; Waters) with an UltiMate 3000 HPLC system at a flow rate of 200 µL min^−1^. For MS evaluation, 30 fractions were sequentially resuspended in 0.1% formic acid (FA), and an LTQ Orbitrap Velos mass spectrometer (Thermo Finnigan, San Jose, CA) coupled on‐line to a Proxeon Easy‐nLC 1000 was used for analysis. Peptides were first loaded onto a trap column (75 µm × 2 cm, Acclaim PepMap100 C18 column, 3 µm, 100 Å; Dionex, Sunnyvale, CA) at a flow rate of 10 µL min^−1^ and then transferred to a reverse‐phase microcapillary column (75 µm × 25 cm, Acclaim PepMap RSLC C18 column, 2 µm, 100 Å; Dionex) at a flow rate of 300 nL min^−1^. HPLC solvent A consisted of 0.1% FA, and solvent B consisted of 100% ACN and 0.1% FA. A 205 min linear gradient (3% to 8% buffer B for 3 min, 8% to 29% buffer B for 176 min, 29% to 41% buffer B for 15 min, 41% to 90% buffer B for 1 min, and 90% buffer B for 10 min) was used for protein identification and quantification. GO analysis of gene enrichment was determined using the database for annotation, visualization, and integrated discovery. The RNA‐seq data were deposited in the SRA database under SRA accession number PRJNA495734. The mass spectrometry proteomics data were deposited with the ProteomeXchange Consortium via the Pride partner repository under accession code PXD010397. For proteomics data accession, Username: reviewer59050@ebi.ac.uk, Password: ILixA4IU can be used.

*Confocal Microscopy*: Oocytes and early embryos were fixed in 4% PBS mixed with paraformaldehyde for 30 min. Oocytes were blocked in 1% BSA dissolved in PBS and incubated with primary antibodies diluted in blocking solution for 1 h. Oocytes/embryos were incubated with secondary antibodies for 30 min after several washes and counterstained with 5 µg mL^−1^ DAPI (4′,6‐diamidino‐2‐phenylindole, Life Technologies) for 10 min. After mounting, oocytes/embryos were examined with a confocal laser‐scanning microscope (Zeiss LSM 780, Carl Zeiss AG, Germany). The antibodies used in these experiments are shown in Table S3 in the Supporting Information.

*Histological Analysis*: Paraffin‐embedded ovary samples were fixed in 10% formalin overnight at 4 °C, deparaffinized, sectioned at a thickness of 5 µm, and stained with hematoxylin and eosin. Images were obtained under an optical microscope.

*Western Blot Analysis*: For total protein extraction, 100 oocytes or embryos were lysed and separated by SDS PAGE and transferred to a PVDF membrane (Millipore). The membrane was incubated with primary antibody followed by HRP‐conjugated secondary antibody, and bands were examined using an enhanced chemiluminescence detection kit (Bio‐Rad). The antibodies used are shown in Table S3 in the Supporting Information.

*Cell Culture, Plasmid Transfection, and Luciferase Assay*: DMEM/high glucose (HyClone, SH30243.01B) supplemented with 10% fetal bovine serum (FBS, BI, 04‐001‐1ACS) was used for HEK293 cell growth, and cells were incubated at 37 °C with 5% CO_2_. The 3'‐UTR sequences of different genes containing IMP2 binding sites were amplified by PCR and cloned into the pEZXGA01 vector (Genecopoeia, Guangzhou, China) in front of the Gaussia luciferase gene. Transient plasmid transfections into cells were performed with the IMP2 expression vector (IMP2‐pCDH) or control vector (pCDH) using the X‐treme‐GENE HP DNA transfection reagent (Roche, Penzberg, Germany). To measure the transfection efficiency, Gaussia luciferase activity was normalized to secreted alkaline phosphatase luciferase activity. The activity ratio was compared with empty vectors, the values of which were arbitrarily set at 1.0. The supernatant from cultured HEK293 cells was collected after 48 h of transfection, and luciferase activity was measured using the Dual Luciferase System (Genecopoeia, #SPGA‐G010) according to the manufacturer's instructions. To validate the direct relationship between IMP2 and IGF2, the human IGF2 L3 5′‐UTR were cloned, and the dual luciferase reporter assay (Genecopoeia, #SPGA‐G010) was performed in vitro in HEK293 cells in human. The 5′‐UTR sequence of IGF2 were amplified by PCR and cloned into the pEZXGA02 vector (Genecopoeia, Guangzhou, China) in front of the Gaussia luciferase gene.

*EU Incorporation Assays*: EU corporation assays were performed as described previously^27^ by using Click‐iT RNA imaging kits (C10329, Invitrogen). Briefly, 2‐cell‐stage embryos from control and *Imp2^−/−^* females were collected. More than 10 embryos were used for each genotype per replicate. Both control and *Imp2^−/−^* females‐derived embryos were incubated in culture medium supplemented with 1 × 10^−3^ m 5‐EU (ethynyl uridine) for 3 h prior to Hoechst 33342 staining. Fixation, permeablization, and staining were performed according to the kit's instructions. The EU fluorescence signal is indicative of total RNA synthesis. Fluorescence signal was calculated as the average intensity after background subtraction. Image J software was used to quantitate the intensity of EU signal. DNA signals were used as the internal reference.

*Detection of Protein Synthesis*: A protein synthesis assay was performed as described previously^43^ using the Click‐iT protein synthesis assay kit (C10428, Life Technologies). Control and *Imp2^−/−^*, 2‐cell‐stage embryos were incubated in culture medium supplemented with 50 × 10^−6^ m HPG (L‐homopropargylglycine, a methionine analog that is incorporated into nascent proteins during active protein synthesis) for 2 h. Embryos were incubated at 37 °C with 5% CO_2_ for 30 min and then washed with PBS. Formaldehyde (3.7%) was used for fixation followed by permeabilization with 0.5% Triton X‐100 for 30 min at room temperature by following the manufacturer instruction. The HPG signal is indicative of the overall level of translation in 2‐cell‐stage embryos. The 2‐cell‐stage embryos were visualized by using confocal microscopy. Fluorescence signal was calculated as the average intensity after background subtraction. HPG signal images were quantified by Image J software (http://rsbweb.nih.gov/ij/). DNA signals were used as the internal reference. Experiments were performed three times with less than 10, 2‐cell‐stage embryos per experiment.

## Conflict of Interest

The authors declare no conflict of interest.

## Supporting information

Supplementary

Supplementary

Supplementary

Supplementary
