# Supplementary material for: RNA‐Binding Protein IGF2BP2/IMP2 is a Critical Maternal Activator in Early Zygotic Genome Activation
Source: Adv Sci (Weinh). 2019 May 24;6(15):1900295. doi: 10.1002/advs.201900295 (PMC6685478; doi:10.1002/advs.201900295)
Supplement: Supplementary file 1 — Supplementary [file ADVS-6-1900295-s001.pdf]

Copyright WILEY-VCH Verlag GmbH & Co. KGaA, 69469 Weinheim, Germany, 2019.

ADVANCED SCIENCE
Open Access

Supporting Information

for *Adv. Sci.,* DOI: 10.1002/advs.201900295

RNA-Binding Protein IGF2BP2/IMP2 is a Critical Maternal Activator in Early Zygotic Genome Activation

*Hong-Bin Liu, Tahir Muhammad, Yueshuai Guo, Meng-Jing Li, Qian-Qian Sha, Chuan-Xin Zhang, Hui Liu, Shi-Gang Zhao, Han Zhao, Hao Zhang, Yan-Zhi Du, Kang Sun, Kui Liu, Gang Lu, Xue-Jiang Guo, Jiahao Sha, Heng-Yu Fan, Fei Gao, and Zi-Jiang Chen**

Copyright WILEY-VCH Verlag GmbH & Co. KGaA, 69469 Weinheim, Germany, 2019.

Supporting Information

RNA-Binding Protein IGF2BP2/IMP2 is a Critical Maternal Activator in Early Zygotic Genome Activation

Hong-Bin Liu, Tahir Muhammad, Yueshuai Guo, Meng-Jing Li, Qian-Qian Sha, Chuan-Xin Zhang, Hui Liu, Shi-Gang Zhao, Han Zhao, Hao Zhang, Yan-Zhi Du, Kang Sun, Kui Liu, Gang Lu, Xue-Jiang Guo, Jiahao Sha, Heng-Yu Fan, Fei Gao, Zi-Jiang Chen

Supporting Information

RNA-Binding Protein IGF2BP2/IMP2 is a Critical Maternal Activator in Early Zygotic Genome Activation

*Hong-Bin Liu, Tahir Muhammad, Yueshuai Guo, Meng-Jing Li, Qian-Qian Sha, Chuan-Xin Zhang, Hui Liu, Shi-Gang Zhao, Han Zhao, Hao Zhang, Yan-Zhi Du, Kang Sun, Kui Liu, Gang Lu, Xue-Jiang Guo, Jiahao Sha, Heng-Yu Fan, Fei Gao, Zi-Jiang Chen**

These authors contributed equally: Hong-Bin Liu, Tahir Muhammad, and Yueshuai Guo

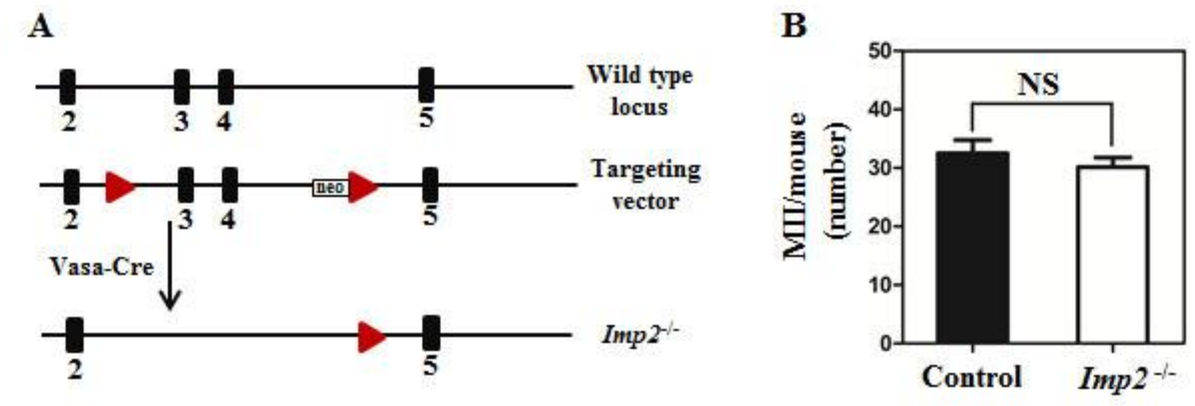

Figure S1. Establishing the *Imp2*$^{-/-}$ mouse and oogenesis.

(A) Schematic diagram showing the gene-targeting vector for creating the conditional *Imp2*-knockout mouse. Loxp recombination sites (red triangles) along with a flanked neomycin-selection cassette were introduced flanking exon 3.

(B) MII oocytes were recovered from hormonally stimulated control (n = 10) and *Imp2*$^{-/-}$ (n = 10) females at 16 h after hCG administration. NS, no statistical difference in Student's *t*-test, $p > 0.05$.

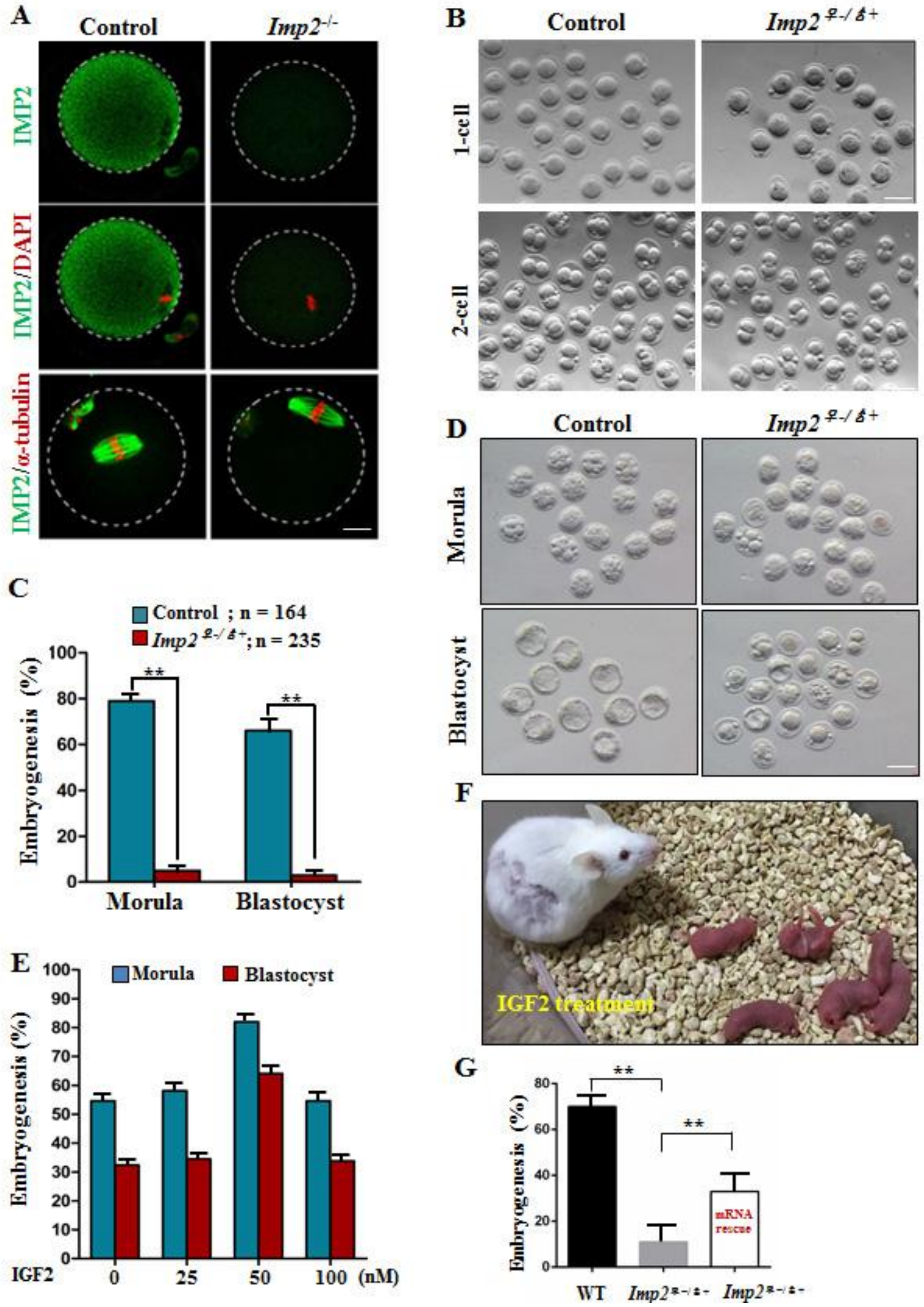

Figure S2. ***Imp2*** **is dispensable for fertilization but is essential for blastocyst development.** **(A)** Immunofluorescence results for control and *Imp2*$^{-/-}$ MII oocytes. Dashed circles represent the outlines of the oocytes. Scale bar, 10 μm.

(B) After hormonal stimulation and *in vivo* mating with wild-type males, 1-cell and 2-cell-stage embryos were flushed from control and *Imp2*$^{-/-}$ female oviducts at embryonic days 0.5 and 1.5. n > 5 mice for each genotype. Scale bar, 100 µm.

(C) Deletion of maternal *Imp2* causes impaired morula and blastocyst formation in *Imp2*$^{-/-}$ females *in vitro*. n > 7 mice for each genotype. Error bars showing the SEM. $**p < 0.01$, Student's *t*-test.

(D) Morphology of embryos collected from the uteri of control and *Imp2*$^{-/-}$ female mice at embryonic days 2.5 and 3.5 after successful mating with adult WT males. Scale bar, 100 µm. n > 6 for both genotypes.

(E) Quantification showing that the 50 nM IGF2 concentration is optimum for early embryonic development. n > 10 mice were used. Error bars indicate the SEM.

(F) Photographs showing the healthy pups delivered by the IGF2 treatment group after embryo transfer.

(G) Representing graph of blastocyst developing from zygotes that had been microinjected with *Imp2* mRNA in *Imp2*$^{-/-}$ females-derived zygotes. WT zygotes used as positive control. Error bars showing the SEM. $**p < 0.01$, Student's *t*-test.

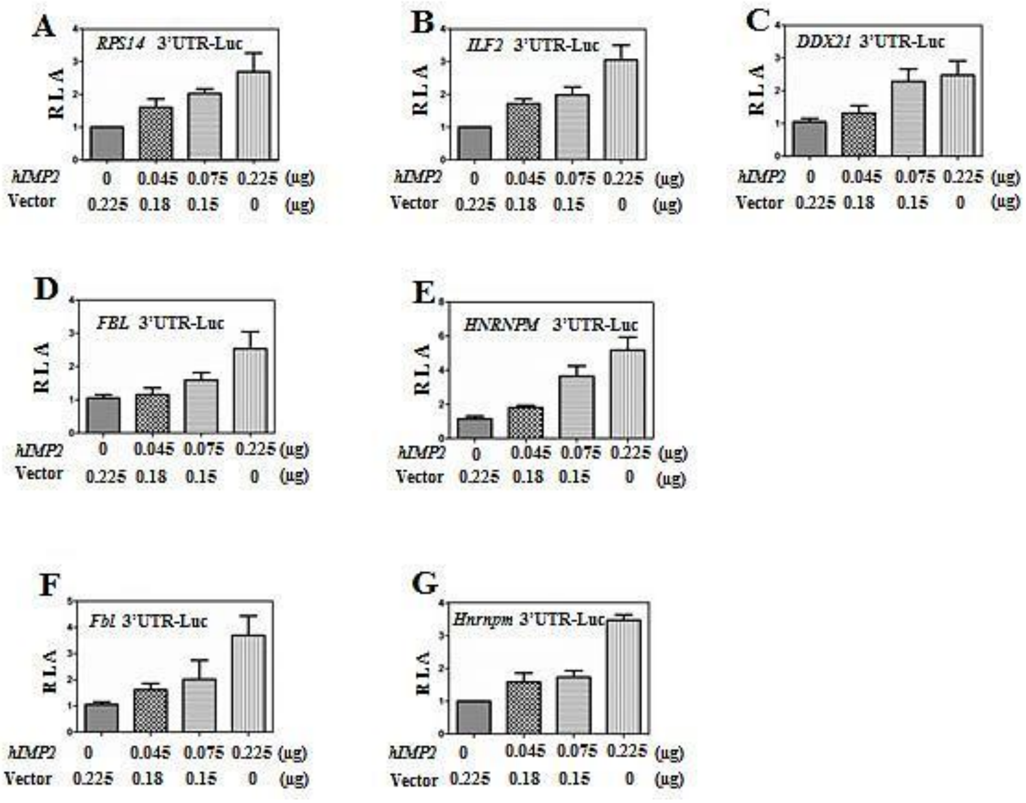

Figure S3. Luciferase reporter activity of downregulated genes.

(A–E) Luciferase activity of indicated downstream genes for RPS14 **(A)**, ILF2 **(B)**, DDX21 **(C)**, FBL **(D)**, and HNRNPM **(E)** in response to *IMP2* in human. Error bars indicate the SEM. **(F** and **G)** Luciferase activity of *Fbl* **(F)** and *Hnrnpm* **(G)** downstream genes in response to *IMP2* in mice. Error bars indicate the SEM. Each value represents the mean of at least three independent experiments.

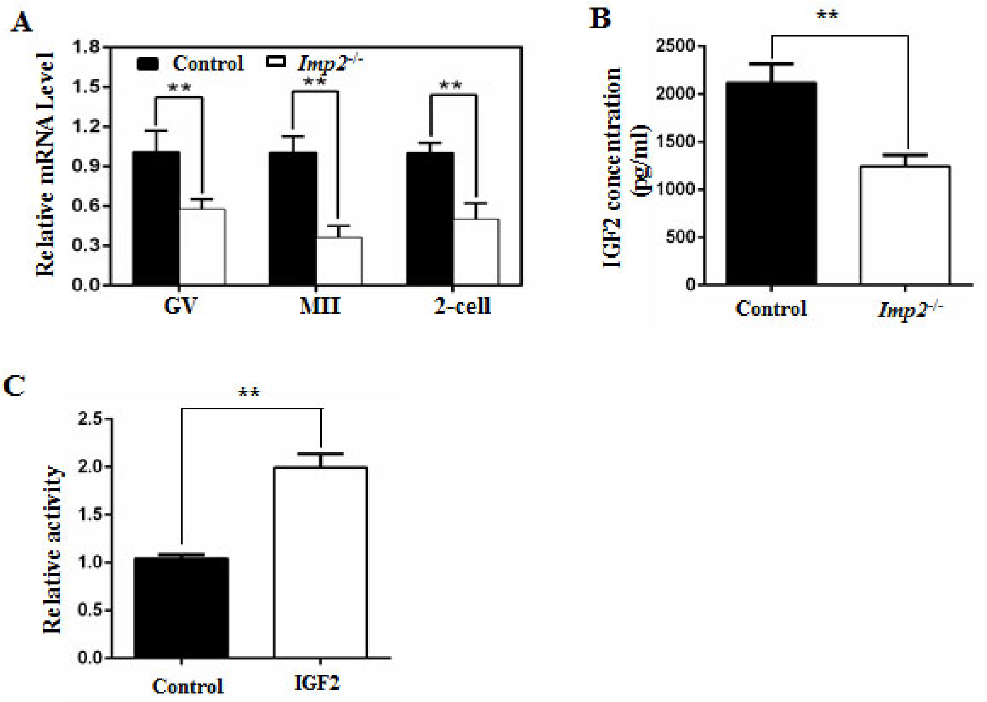

Figure S4. Direct relationship of IMP2 with IGF2.

(A) qPCR results showing mRNA level of *IGF2* in GV-stage, MII-stage oocytes and 2-cell-stage embryos-derived from control and *Imp2*$^{-/-}$ female**.** Error bars showing the SEM. $**p < 0.01$, Student's *t*-test.

(B) IGF2 concentrations in culture medium containing GV-stage oocytes of different genotypes. GV-stage oocytes derived from control and *Imp2*$^{-/-}$ female mice were cultured in M16 medium supplemented with 2 μM milrinone for 12 h. Error bars showing the SEM. $**p < 0.01$, Student's *t*-test.

(C) In human HEK293 cells, IMP2 enhanced the luciferase activity of IGF2 by 2-fold compared with negative control. Error bars showing the SEM. $**p < 0.01$, Student's *t*-test.

Table S1. Gene description.

Gene Symbol	Species	Gene name	Transcription binding sites
CCAR1	Human	cell division cycle and apoptosis regulator 1	CATCTCCTACCATCTATACACAG
Ccar1	mouse	cell division cycle and apoptosis regulator 1	CATCTCCTACCATTTATACCCAGCAG
FBL	human	Fibrillarin	AAGAGGACCAACATCATTCCTGTGATCGAGGA
Fbl	mouse	Fibrillarin	AAGAGGACTAACATTATTCCTGTAATTGA
HNRNPM	human	Heterogeneous nuclear ribonucleoprotein M	CCAAGGCCTCTCTCAATGGGGCTGATA
Hnrnpm	mouse	heterogeneous nuclear ribonucleoprotein M	CCAAGGCCTCACTCAATGGGGCTGACA
FYTTD1	human	forty-two-three domain containing 1	CTTTTCACATAGGCCAAGAACTCATTGCAAAA
ILF2	human	interleukin enhancer binding factor 2	CCATCCATCTATAATGGAGGATACCAACATT
RPS14	human	ribosomal protein S14	TCACCCCACAAACACACACCAAG
DDX21	human	DEXD-box helicase 21	CTTCACTTAAATTATTTCATCTG
RPL32	human	Ribosomal protein L32	TACATAGGTCATGAGGGATATG
PHGDH	human	phosphoglycerate dehydrogenase	AGGGAGAGAAAATCCACACTCTTGGGCTGAAC
PSAT1	human	phosphoserine aminotransferase 1	TTAAATCACCCTATCCTTTG
HNRNPA2B1	human	heterogeneous nuclear ribonucleoprotein A2/B1	ATTATTTAACAACATTACCTTACTG

Table S2. Primer Sequences.

Gene name	Primers Sequence	Uses
Ddx21	F: 5’-TGATGTCCGAACTGAAGCAG-3’<br>R: 5’-TCGATATCCGTCTGGAGGTC-3’	Real-time
Ccar1	F: 5’-CCAAAACCAAAACGGAGAAA-3’<br>R: 5’-TTCCTCCTCCTCCCTATCGT-3’	
Hnrnpm	F: 5’-GCTGGAAGACTTGGAAGCAC-3’<br>R: 5’-TCACAATGCCTATTCCACGA-3’	
Ilf2	F: 5’-ATTCTGGCTGCAGGACTGTT-3’<br>R: 5’-AAGCCTCCATGGGAGAGAAT-3’	
Fyttd1	F: 5’-AGACACTCGTCAGGCAACCT-3’<br>R: 5’-ATTGACGCGTTCTCTTTGCT-3’	
Rpl32	F: 5’-AACCCAGAGGCATTGACAAC-3’<br>R: 5’-ATTGTGGACCAGGAACTTGC-3’	
Rps14	F: 5’-CAAGGGGAAGGAAAAGAAGG-3’<br>R: 5’-GAGGACTCATCTCGGTCAGC-3’	
Gtf2i	F: 5’-CCTGCCGAAGATGAAGAGTC-3’<br>R: 5’-TTCGGTTCCAACAACAAACA-3’	
Mpc2	F: 5’-TGTTGCTGCCAAAGAAATTG-3’<br>R: 5’-GCTAGTCCAGCACACACCAA-3’	
Set	F: 5’-CACGAAGAGCCAGAGAGCTT-3’<br>R: 5’-CATGTCGGGAACCAGGTAGT-3’	
Sf1	F: 5’-AGCTAGGGGAAGCTCCTGTC-3’<br>R: 5’-GGCGGCTCTGAGTTGTAGAC-3’	
Pgrmc1	F: 5’-TTTTGCCTGGACAAAGAAGC-3’<br>R: 5’-TCCGAGCTGTCTCGTCTTTT-3’	
Nat10	F: 5’- AGCCATTTCCCGCTTGTACT -3’<br>R: 5’- CCTGAGGGCAGCTCAATCTC -3’	
Rps19	F: 5’-TACACACGAGCTGCTTCCAC-3’<br>R: 5’-CTGGGTCTGACACCGTTTCT-3’	
Usp10	F: 5’-GTCGAGCCTGTCTGAAAAGG-3’<br>R: 5’-GTGTCTTCCAGCTCCTCGTC-3’	
Mrpl4	F: 5’-GAGATGCCCAAGAATGTCGT-3’<br>R: 5’-CCTGCCAGAGTAGCTTGTCC-3’	
Dnttip2	F: 5’-AACTGACAGCCCAAAACCAC-3’<br>R: 5’-ACTGCTGAAGGCTGGTGTCT-3’	
Nelfe	F: 5’-TCTGAAGAAGCAGAGCAGCA-3’<br>R: 5’-ACCAGTTGTTTGGCCTGTTC-3’	
Hnrnpl	F: 5’-GAAGCTGACCTTGTGGAAGC-3’<br>R: 5’-CCGGCAATGTAGATCTGGTT-3’	
Fbl	F: 5’-TGGTCTGGTCTACGCAGTTG-3’<br>R: 5’-GGGTGTCGAGCATCTTCAAT-3’	
Phgdh	F: 5’- GGAGGCTTTCCAGTTCTGCT -3’<br>R: 5’- CTGCGATCCCCTCTCCCTAT -3’	
Ccar1	F: 5’ CCAGCAAACTATCAGTTAA-3’	siRNA

	R: 5’CCAGTCAACAGCAAACTCA-3’	
Rps14	F: 5’ TGGAGACGACGATCAGAAA-3’<br>R: 5’ TCACTGCCCTGCACATCAA-3’	
Imp2 +flox-62	F: 5’-CAGCCCCGAGTGAGGAGAGTAGC-3’<br>R: 5’-CCCCCATCGACCCCCAGTTT-3’	Genotyping
Imp2 ∆-50	F: 5’-CAATACTTCTGGACTTTTCA-3’<br>R: 5’-CTTTTCCTGGAGACTTTATG-3’	

Table S3. Antibody information.

**Protein name**	**Manufacture (catalogue number)**	**Applications (working dilution)**
IGF2BP2	Cell signaling (14672)	WB(1:1000)
IGF2BP2	Abcam (ab124930)	IF(1:250)
CCAR1	Gentex (GTX110892)	WB(1:200)
FBL	Abcam (ab166630)	WB(1:250)
RPS14	Proteintech (16683-1-AP)	WB(1:100)
DDX21	Santa cruz (sc-376785)	WB(1:50)
ILF2	Abcam (ab154169)	WB(1:300)
ACTIN	Cell signaling (4970)	WB(1:1000)
ERK1/2	Santa Cruz (sc-94)	WB(1:1000)

Table S4. RNA seq and protein merged analysis.

NO	Gene Name	Read count KO	Read count WT	log2.Fold	CLIP binding	Expression status
1	*Parp1*	81.50	166.66	-1.03	Y	Down-regulated
2	*Mrpl4*	44.11	112.39	-1.35	Y	Down-regulated
3	*Actn2*	4.62	25.07	-2.44		Down-regulated
4	*Dnttip2*	77.01	387.65	-2.33		Down-regulated
5	*Ilf2*	133.52	357.82	-1.42	Y	Down-regulated
6	*Gtf2i*	20.89	79.69	-1.93	Y	Down-regulated
7	*Nelfe*	17.28	51.36	-1.57		Down-regulated
8	*Fyttd1*	21.75	47.88	-1.14	Y	Down-regulated
9	*Usp10*	173.27	348.32	-1.01	Y	Down-regulated
10	*Mpc2*	39.26	108.95	-1.47		Down-regulated
11	*Hnrnpm*	67.83	268.41	-1.98		Down-regulated
12	*Sntb2*	25.65	75.90	-1.57	Y	Down-regulated
13	*Rps14*	30.04	79.59	-1.41	Y	Down-regulated
14	*Psat1*	81.21	368.99	-2.18	Y	Down-regulated
15	*Syncrip*	31.52	96.54	-1.61		Down-regulated
16	*Ddx21*	50.57	232.77	-2.20	Y	Down-regulated
17	*Ccar1*	223.32	557.25	-1.32	Y	Down-regulated
18	*Hnrnpl*	47.62	103.81	-1.12	Y	Down-regulated
19	*Gatad2b*	62.64	131.51	-1.07		Down-regulated
20	*Rps19*	21.42	65.12	-1.60		Down-regulated
21	*Hnrnpa2b1*	310.34	630.17	-1.02	Y	Down-regulated
22	*Set*	347.04	998.66	-1.52		Down-regulated
23	*Rpl32*	29.49	70.05	-1.25	Y	Down-regulated
24	*Sf1*	90.16	190.67	-1.08	Y	Down-regulated
25	*Pgrmc1*	34.41	75.15	-1.13	Y	Down-regulated
26	*Phgdh*	15.38	76.50	-2.31	Y	Down-regulated
27	*Rpl23*	28.63	71.64	-1.32		Down-regulated
28	*Fbl*	58.81	163.32	-1.47	Y	Down-regulated
29	*Nat10*	37.06	127.43	-1.78		Down-regulated
30	*Gls*	35.29	118.00	-1.74	Y	Down-regulated
31	*Rbm10*	90.19	256.81	-1.51	Y	Down-regulated
32	*Gar1*	27.29	82.57	-1.60		Down-regulated
33	*Cit*	118.99	42.17	1.50		Up-regulated
34	*Degs1*	322.77	95.51	1.76		Up-regulated

Table S5. Human IGF2 binding sites (L3)

Binding site	Location	Sequence
#1	chr11:2159633-2159699	AAAAGTACAACATCTGGCCCGCCCCAGCCCGAAGAC AGCCCGTCCTCCCTGGACAATCAGACGAATT
#2	chr11:2160385-2160454	TCCCGGGCGCCGTCCGCGGGGTCGCGCTCCGCCGGGC CTGCGGATTCCCCGCCGCCTCCTCTTCATCTAC
#3	chr11:2160478-2160514	CCTCCATCGGGCAAGGCGGCCCCGCGTCGACGCCGCC

Table S6. List of ZGA relevant genes after merging 2-cell-stage embryos proteomic data compared with published PAR-CLIP data

No	Gene Name
1	*Ilf2*
2	*Ptms*
3	*Gnb2*
4	*Snx5*
5	*Rps14*
6	*Ptges3*
7	*Hplbp3*
8	*Psat1*
9	*Rpl27a*
10	*Rpl31*
11	*Cbx3*
12	*Idha*
13	*Rpl38*
14	*Enah*
15	*Prkaca*
16	*Rbm10*
17	*Rpl28*
18	*Tagn2*
19	*Hnrnph3*
20	*Hk1*
21	*Pls3*
22	*Bag3*
